# Upregulation of the zebrafish Nogo-A homologue, Rtn4b, in retinal ganglion cells is functionally involved in axon regeneration

**DOI:** 10.1186/s13064-015-0034-x

**Published:** 2015-03-20

**Authors:** Cornelia Welte, Sarah Engel, Claudia A O Stuermer

**Affiliations:** Department of Biology, University of Konstanz, Universitätsstraße 10, 78457 Konstanz, Germany

**Keywords:** Optic nerve lesion, Axon regeneration, Rtn4b upregulation, ER, Morpholino downregulation, Retinal ganglion cells, Neuron-intrinsic determinants, Nogo-A/RTN4A

## Abstract

**Background:**

In contrast to mammals, zebrafish successfully regenerate retinal ganglion cell (RGC) axons after optic nerve section (ONS). This difference is explained on the one hand by neurite growth inhibitors in mammals (including Nogo-A), as opposed to growth-promoting glial cells in the fish visual pathway, and on the other hand by the neuron-intrinsic properties allowing the upregulation of growth-associated proteins in fish RGCs but not in mammals.

**Results:**

Here, we report that Rtn4b, the zebrafish homologue of mammalian Nogo-A/RTN4-A, is upregulated in axotomized zebrafish RGCs and is primarily associated with the endoplasmic reticulum (ER). Rtn4b functions as a neuron-intrinsic determinant for axon regeneration, as was shown by downregulating Rtn4b through retrogradely transported morpholinos (MOs), applied to the optic nerve at the time of ONS. MO1 and MO2 reduced the number of axons from retina explants in a concentration-dependent manner. With MO1, the reduction was 55% (70 μM MO1) and 74% (140 μM MO1), respectively, with MO2: 59% (70 μM MO2) and 73% (140 μM MO2), respectively (compared to the control MO-treated side). Moreover, regenerating axons 7d after ONS and MO1 or MO2 application were labeled by Alexa488, applied distal to the first lesion. The number of Alexa488 labeled RGCs, containing the Rtn4b MO1 or MO2, was reduced by 54% and 62%, respectively, over control MO.

**Conclusions:**

Thus, Rtn4b is an important neuron-intrinsic component and required for the success of axon regeneration in the zebrafish visual system. The spontaneous lesion-induced upregulation of Rtn4b in fish correlates with an increase in ER, soma size, biosynthetic activity, and thus growth and predicts that mammalian neurons require the same upregulation in order to successfully regenerate RGC axons.

## Background

The visual pathway in teleost fish is well-known for the capacity of the retinal ganglion cells (RGCs) to regenerate axons following optic nerve section (ONS) and to restore function [1]. In contrast, CNS axons in mammals do not spontaneously regenerate because of inhibitory properties of the glial cells in the environment of lesioned axons [2] and the poor neuron-intrinsic properties [3,4]. In fish, the glial cell environment is apparently growth-permissive [5,6]. Moreover, fish RGCs possess the unique ability to spontaneously activate the cellular machinery required for axon regrowth including upregulation of growth-associated proteins [7,8] and rise in the neuron’s biosynthetic activity.

One of the strongest oligodendrocyte and myelin-associated inhibitors in mammals is Nogo-A [2]. More than 95% of Nogo-A is localized at the endoplasmic reticulum (ER) [9,10], and only small amounts emerge at the surface [2]. Nogo-A is also expressed in neurons, particularly in those with far-projecting axons. Neuronal Nogo-A was reported to negatively regulate neuronal plasticity [11,12], but a positive influence on neurite extension has also been observed. Nogo-A promotes sprouting of axons in the lesioned mouse optic nerve and regeneration in RGCs with elevated Nogo-A expression levels [13,14].

In zebrafish embryos, the Nogo-A homologue Rtn4b [15] was discovered in many differentiating brain regions [16] including the developing RGCs, and its downregulation caused severe defects in the retinotectal projection.

Here, we asked whether Rtn4b would be upregulated in adult fish RGCs after ONS and promote axon regeneration. This can be assessed by the *in vivo* application of specific morpholinos (MOs) to the eye-side stump of the lesioned optic nerve as done with reggie-1 and −2, which massively impaired axon regeneration [17]. Our results indeed show that zebrafish RGCs require Rtn4b as a neuron-intrinsic determinant of axon regeneration.

## Results

### Rtn4b expression in zebrafish RGCs and upregulation after optic nerve lesion

**Figure 1:**
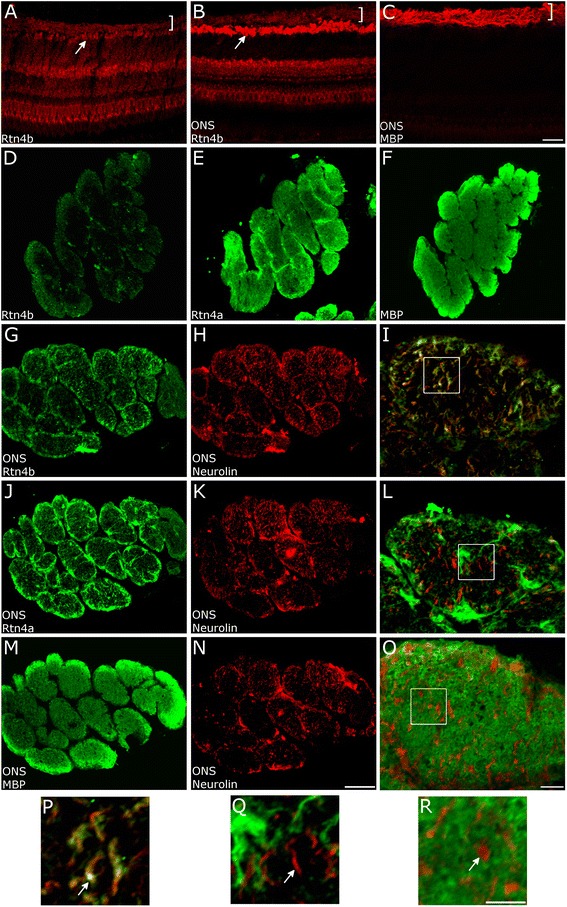
**Expression pattern of Rtn4b in the zebrafish retina and optic nerve.** Cross sections of the zebrafish retina normal **(A)** and 10 days after ONS **(B,C)** were exposed to ABs against Rtn4b (A,B) and MBP (C). Weak Rtn4b staining is seen across all retinal layers including RGCs (white arrow) in the normal retina (A). RGCs robustly upregulate Rtn4b 10 days after ONS (B). The RGC axons in the retina on top of the RGCs (bracket) are also weakly labeled but are more intensely stained by the AB against MBP (C). Scale bar, 50 μm. Cross sections through the normal zebrafish optic nerve **(D,E,F)** show very weak labeling with Rtn4b AB (D). (E) Rtn4a AB stains across the normal nerve similar to MBP. Labeling with the MBP AB is strong in the normal nerve (F) as well as at 10 days after ONS **(M)**. Scale bar, 10 μm. Rtn4b AB in nerves at 10 days after ONS **(G,I)** labels portions of RGC axons identified as axonal (rather than glial) by the AB against neurolin **(H)**. (I), overlay. Scale bar, 20 μm. **(J)** Rtn4a AB in the10-days ONS nerve stains, in particular, the boundaries of fascicles and subdivisions of the fascicles. **(K,L)** The neurolin-positive regenerating RGC axons are not labeled with Rtn4a to any significant extent, in contrast to Rtn4b labeling (I). (M) MBP staining is intense in the 10-day ONS nerve. **(N,O)** Neurolin-positive regenerating RGC axons are located amidst the myelin. Boxed areas in (I,L,O) are enlarged in **(P,Q,R)**. Arrows point to neurolin-positive axons which are also Rtn4b AB-positive (P), but seem not significantly co-localized with Rtn4a (Q), and lie amidst the MBP labeling of myelin (R). Scale bar, 10 μm.

**Figure 2:**
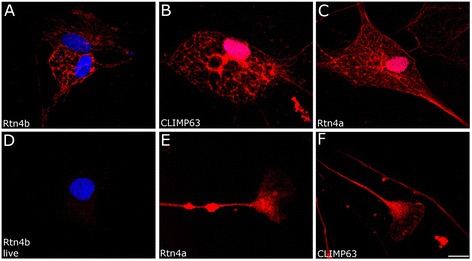
**Rtn4a and Rtn4b staining of ER in zebrafish oligodendrocytes. (A)** Labeling of fixed zebrafish oligodendrocytes with Rtn4b reveals a reticular structure similar to the ER staining with CLIMP63 AB **(B). (C)** Rtn4a AB also labels ER in oligodendrocytes. **(D)** Exposing live oligodendrocytes to Rtn4b AB gives no cell surface staining. DAPI stains the nuclei in (A,D) and also in (B,C) where, however, the red ER stain covers the blue. **(E)** Zebrafish RGC axons and growth cones are labeled by the Rtn4b AB. These structures are also labeled with CLIMP63 AB **(F)**. Scale bar, 10 μm.

The affinity purified antiserum against zebrafish Rtn4b [16] labeled all retinal layers but was brighter over RGC somata compared to other retinal neurons (Figure 1A). The RGC axon layer which was intensely labeled by the anti-MBP antibody (AB) (fish RGC axons are myelinated in their intraretinal path) was only weakly stained by the Rtn4b AB (Figure 1A, B, C). Ten days after ONS, RGC somata had significantly increased expression of Rtn4b indicating that ONS leads to Rtn4b upregulation in neurons (Figure 1B). In the normal optic nerve, Rtn4b labeling was weak (Figure 1D) whereas anti-MBP AB strongly labeled the myelin (Figure 1F, M) in the normal nerve and after ONS. The staining with Rtn4a AB was similar to MBP, but the AB labeled in addition the boundaries of axon fascicles and further subdivisions of the fascicles (Figure 1E). Rtn4a therefore appears to reside in astrocytic structures as suggested earlier [18] and myelin. In the nerve 10 days after ONS, Rtn4b labeling was associated with glial cell processes around fascicles and more strikingly with regenerating RGC axons which were identified by anti-neurolin AB [19] (Figure 1G, H, I, P). Accordingly, axons and growth cones in culture were also labeled (Figure 2E). Rtn4a AB also stains RGC growth cones *in vitro* [18] but in sections through the nerve strongly stained the fascicle boundaries and subdivisions rather than neurolin-positive regenerating axons (Figure 1J, K, L, Q). In the nerve 10 days after ONS, myelin detected by MBP AB was intense and the neurolin-positive regenerating axons were located amidst the myelin staining (Figure 1M, N, O, R). Together, this staining shows that regenerating RGC axons in the nerve and *in vitro* are Rtn4b-positive and cross through MBP-labeled myelin. Rtn4a is in myelin and astrocytic fascicle boundaries and subdivisions but not to the same extent in neurolin-positive axons as Rtn4b. Rtn4b appears less prominent in CNS myelin in the retina and optic nerve but is significantly upregulated in RGCs and RGC axons after ONS.

To clarify whether Rtn4b in fish is, as in mammals, localized at the ER, we exposed zebrafish oligodendrocytes *in vitro* to Rtn4b AB and the AB against CLIMP63 specific for the ER. Rtn4a AB was used for comparison. Rtn4b as well as Rtn4a labeled a reticular network that is positive for CLIMP63 AB and reached into the cellular processes (Figure 2A, B, C) like Nogo-A in mammals [9]. Growth cones of RGC axons showed Rtn4b and CLIMP63 staining (Figure 2E, F), suggesting that the ER extends into the tips of elongating axons. We also tested if Rtn4b might be visible at the cell surface as reported for Nogo-A in mammalian oligodendrocytes and neurons [2]. We were not able to detect cell surface staining by exposing live cells to Rtn4b AB, either because the protein does not reach the surface or in amounts that are too small to be detected by the present staining procedure (Figure 2D).

**Figure 3:**
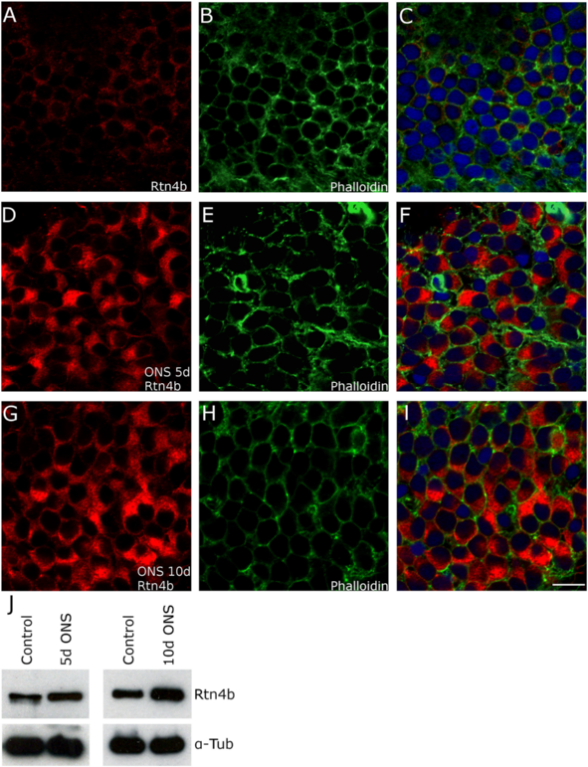
**Upregulation of Rtn4b in zebrafish RGCs after ONS.** RGCs in retina whole mounts showed weak immunostainings in the cytoplasm after exposure to Rtn4b AB **(A). (B,E,H)** Labeling with Phalloidin against F-actin shows all cells and their cytoplasm. **(C,F,I)** Merge of (A,B), (C,D), and (G,H) with DAPI stainings to visualize nuclei. **(D,E,F)** 5 days after ONS, the RGCs exhibit a significant increase in size and increase in Rtn4b labeling intensity (48% in comparison to control, *P* < 0.01) in the cytoplasm. **(G,H,I)** 10 days after optic nerve sections, the size on the RGCs and the intensity of Rtn4b staining is still highly increased (53% over controls, *P* < 0.01). Scale bar, 10 μm. **(J)** This apparent increase in the 100 kd Rtn4b protein is also seen in Western blots with retinae at 5 (*P* < 0.05) and 10 days (*P* < 0.0001, Student’s *T*-test) after ONS. Anti-alpha tubulin served as loading control.

Next, we analyzed changes in axotomized RGCs in retina whole mounts (Figure 3A, B, C, D, E, F, G, H, I). RGC somata at 5 days after ONS increased in area by 87% (the cell body reaction), and the cytoplasm was filled entirely by Rtn4b AB labeling (Figure 3D) associated with cloudy structures, typical for ER staining with anti-protein disulfide isomerase (PDI) in mammalian cells (ABCAM home page). Rtn4b staining intensity was upregulated in intensity by 48% when compared to controls (*P* < 0.01). Ten days after ONS, Rtn4b protein levels were even more elevated with an increase of 54% in comparison to control (*P* < 0.01).

Quantitative Western blots with retina lysates, control *versus* 5 and 10 days after ONS, also showed a significant upregulation of 100 kd Rtn4b by 45% (5 days; *P* < 0.01) and 58% (10 days; *P* < 0.0001), respectively (Figure 3J).

### Rtn4b is essential for RGC axon regeneration

**Figure 4:**
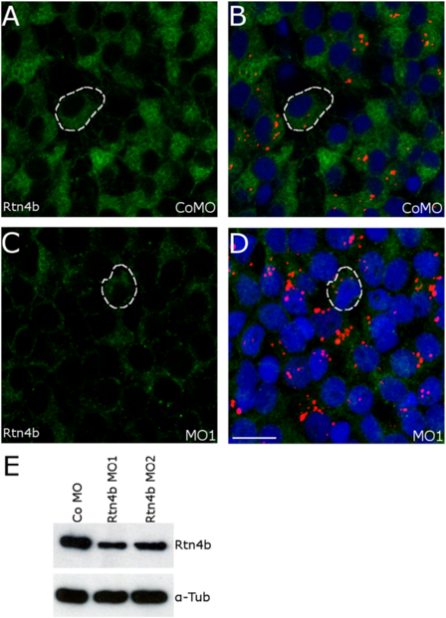
**Downregulation of Rtn4b in RGCs by MO1.** After application of the control (Co) MO to the lesioned optic nerve, Rtn4b labeling of RGCs in retina whole mounts was intense at 5 days after ONS **(A,B)**, but was markedly reduced when MO1 against Rtn4b **(C,D)** was offered. (B,D) The Rtn4b AB-labeled cells contain Lissamine (red) conjugated to the MOs. Examples of RGCs are outlined (white interrupted lines). DAPI stains the nuclei. Scale bar, 10 μm. **(E)** Western blot analysis showing a significant decrease in Rtn4b expression in retinae 5 days after ONS and application of MO1 and MO2 (*P* < 0.0001), respectively, to the optic nerve. Alpha-tubulin served as loading control.

To determine whether Rtn4b is indeed needed for RGC axon regeneration, we downregulated Rtn4b by placing a MO-soaked piece of gel foam directly at the eye-sided optic nerve at the time of ONS [17]. The MOs are retrogradely transported into the RGCs as is demonstrated by using lissamine-labeled MOs. To minimize the danger of potential off-target effects, two different MOs, MO1 and MO2, were used and applied at two different concentrations (either 70 or 140 μM) to the left optic nerve in parallel with control MO application to the right nerve within the same animal. That MO1 and MO2 specifically downregulate Rtn4b in a concentration-dependent manner was demonstrated earlier in zebrafish embryos [16]. Five days after MO1 or MO2 application, the Rtn4b immunostaining intensity in RGCs in whole mounts was markedly reduced (Figure 4C,D), whereas RGCs receiving control MO were intensively labeled (Figure 4A, B). That MO1 and MO2 led indeed to Rtn4b downregulation was confirmed by Western blots of retinae, 5 days after ONS and MO application (Figure 4E) showing an overall significant reduction of Rtn4b by 32% with each MO1 and MO2 (*P* < 0.0001, MO1; *P* < 0.0001, MO2).

### *Ex vivo* outgrowth assay

**Figure 5:**
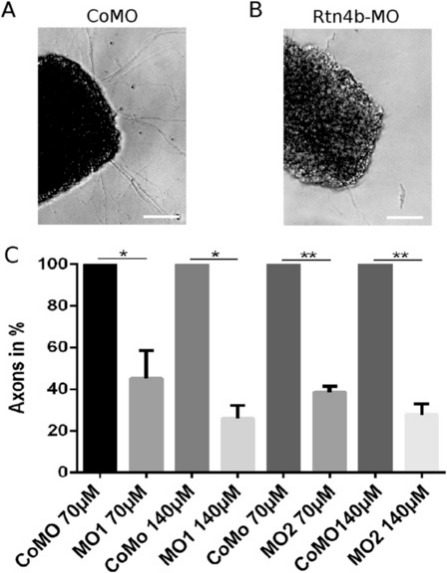
**Quantification of axon number after MO application in the outgrowth assay. (A,B)** Retina mini-explants isolated from retinae 5 days after (A) control MO (Co) or (B) Rtn4b MO1 application to the optic nerve, extend axons and axon fascicles (arrows) after 24 h *in vitro*. Outgrowth is significantly reduced on the Rtn4b MO-treated side. Scale bar, 100 μm. **(C)** The histogram demonstrates the decline in the number of axons extending from retina explants *in vitro* after MO1 and MO2 application to the optic nerve, in comparison to axon number from control (Co) MO-treated fish (100%). Bars indicate standard deviation. The differences between groups are statistically significantly different. Quantification was done on three replicates from three different experiments, and for statistical analysis, Student’s *T*-test was used. *P < 0.05, **P < 0.01.

Next, we analyzed whether Rtn4b MOs impair axon regeneration. In the so-called outgrowth assay, the retinae were divided into mini-explants at 5 days after ONS and MO application and seeded onto pLys-coated coverslips. Counts of axons in Rtn4b MO-treated compared to control MO-treated retinae at 24 h shows that Rtn4b downregulation reduced axon outgrowth significantly. MO1-treated retinae at 70 and 140 μM, respectively, gave a reduction in number of axons of 55% and 74% over controls (Figure 5). MO2-treated retinae at 70 and 140 μM, respectively, showed a 59% and 73% reduction over controls (80 explants were evaluated per experiment). This reduction in axon number was statistically significant with MO1 at 70 μM (*P* < 0.05), with MO2 at 70 μM (*P* < 0.01) and at 140 μM MO1 (*P* < 0.05) and MO2 (*P* < 0.01). Thus, downregulation of Rtn4b with two unrelated MOs blocks RGC axon outgrowth in a concentration-dependent manner.

### *In vivo* regeneration assay

**Figure 6:**
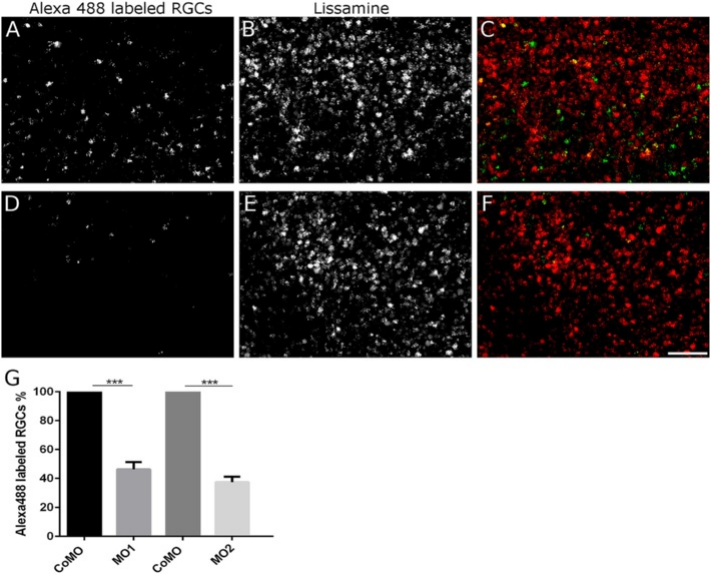
**Rtn4b MO-induced reduction in axon regeneration in the** ***in vivo*** **regeneration assays. (A-F)** After application of Alexa488 to the regenerating axons (distal from the original lesion and MO application site), the retrogradely labeled RGCs are counted in retina whole mounts. Many more Alexa488-labeled RGCs are recognized 9 days after ONS and control (Co) MO application (A) than on the contralateral retina (D) belonging to the nerve that received Rtn4b MO1 (or MO2). (B, E) The RGCs contain lissamine associated with the MOs. (C,F) Merge of (A,B) and (D,E). Scale bar, 50 μm. **(G)** The histogram demonstrates the decline in the number of Alexa-labeled RGCs after MO1 and MO2 application to the optic nerve, in comparison to axon number from control (Co) MO-treated fish (100%). Bars indicate standard deviation. Three different experiments with *n*, 10 retinal squares (300 × 300 μm) for each experimental group were statistically evaluated using Student’s *T*-test. The differences between groups are statistically significantly different, ***P < 0.001.

In a second assay, the optic nerve of fish after ONS and MO treatment was re-sectioned at 7 days, 2 to 3 mm distal from the first lesion, and Alexa488-dextran was applied to retrogradely label RGCs with regenerating axons [17]. Two days later, the dextran-labeled RGCs were counted in left and right retina whole mounts (left side: Rtn4b MO1 and Rtn4b MO2, respectively; right side: control MO) in seven independent experiments (Figure 6A, B, C, D, E, F). The number of dextran-labeled RGCs was on average reduced by 54% over controls with MO1 (*P* < 0.001) and by 62% with MO2 (*P* < 0.001) (Figure 6G). Thus, downregulation of Rtn4b significantly blocks RGC axon regeneration.

## Discussion

This study showed that zebrafish upregulate Rtn4b after ONS and need Rtn4b for axon regeneration. This was demonstrated by employing the MO-mediated downregulation of Rtn4b *in vivo*, with two different MOs and at different concentrations. Moreover, impaired axon regeneration was observed in two independent experimental approaches: the *ex vivo* outgrowth and the *in vivo* regeneration assay. Therefore, we conclude that Rtn4b belongs to the group of growth-associated proteins that axotomized fish RGCs upregulate in order to regenerate axons and that are indicative of the growth-supportive neuron-intrinsic properties of these neurons.

The present technique involving downregulation of specific growth-associated proteins *in vivo* by MO application to lesioned zebrafish CNS fiber tracts has been successfully applied in the past [20,21,17,22]. The danger of potential MO side effects was minimized by employing two different MOs against Rtn4b and different MO concentrations, in parallel with control MO. That MO1 and MO2 specifically target Rtn4b is further supported by Western blots in retinae and embryos. In zebrafish embryos, the appropriate rescues involving co-injection of MO-resistant RNAs partially restored the defects caused by Rtn4b downregulation [16]. Such rescue experiments are not feasible in the present experimental setting since lesioned axons do not take up or retrogradely transport RNAs or vectors, nor can RGCs be transfected by injecting the agents into the vitreous. Still, present and earlier controls together with results from several studies using the *in vivo* MO application to downregulate specific proteins speak for the reliability of our results showing that Rtn4b is essential for axon regeneration.

In a previous study, MO-mediated downregulation of reggie-1 and −2 impaired RGC axon regrowth by up to 70%. Reggies are intracellular membrane-associated proteins involved in Rab11-dependent cargo recycling and trafficking [4,23]. Their downregulation affects the machinery that regulates the delivery of membrane and proteins to the elongating growth cone [24]. This function is essential for neurite elongation and explains why the reduction in axon regeneration was massive with reggie MOs. The present results suggest that Rtn4b is equally important even though the molecular mechanism is unclear (see below).

To get an impression of the significance of a given protein for axon growth, its forced upregulation in mammalian RGCs can be informative. For instance, adeno-associated virus (AAV)-mediated upregulation of reggie-1 in rat RGCs increased the number and length of regenerating axons in the optic nerve [25]. In a similar experiment, AAV-mediated upregulation of Nogo-A enhanced sprouting of lesioned axons in the optic nerve of Nogo-A knockout mice [13,14]. This growth is always minimal compared to fish RGC axons. Likewise, intracellular Nogo-A mildly facilitated neurite formation in mouse midbrain neurons and F11 cells [26,13]. Compared to the situation in mammals, the increase in ER and soma size associated with Rtn4b upregulation in fish RGCs is remarkable and is evidently causally linked to the success of axon regeneration in vertebrates [7].

More than 95% of Nogo-A/RTN4A is associated with the ER where it has been shown to promote the formation of ER tubules [2,10]. Zebrafish Rtn4b is also predominantly localized at the ER as shown by the present immunostainings. The increase in Rtn4b staining in the retina and RGCs is consistent with an expansion of the ER in response to ONS during the upregulation of protein synthesis (cell body response, [8,7]). It is possible that Rtn4b enhances growth through its ER structuring ability [9], or it plays a direct role in the production of specific proteins and the upregulation of growth-associated molecules in regeneration-competent neurons. Rtn4b and CLIMP63 staining reaches into the axons and RGC growth cones which is consistent with the notion that they contain ER and synthesize molecules relevant for growth and guidance [27]. RTN4a as an ER shaping protein is reportedly involved in the redistribution of PDI in superoxide dismutase (SOD)1 dependent amyotrophic lateral sclerosis [28] emphasizing the importance of ER-associated functions of RTN4. Moreover, the ER structure in axons depends on microtubules and GTPases like atlastin-1. Atlastin-1 loss of function inhibits axon elongation [29-31], probably due to impairment of ER structure and distribution. Therefore, it is conceivable that zebrafish Rtn4b subserves similar important functions for the integrity of the ER and growth, including reforming growth cones and axons.

The other functionally relevant location of Nogo-A/RTN4-A in mammals is the cell surface. Growing axons typically are inhibited by Nogo-A exposed on the surface of oligodendrocytes and CNS myelin [2]. Surface-exposed Nogo-A is also known as an inhibitor of neuronal plasticity and regulator of structural integrity of neuronal connections. Whether a fraction of zebrafish Rtn4b is exposed on the cell surface is relevant for experiments testing its function as potential growth inhibitor associated with glial cells and CNS myelin. In mammals, surface-exposed Nogo-A acts as a ligand for two receptor complexes [2,32] connecting to signaling cascades that inhibit axon growth. Whether zebrafish Rtn4b exerts inhibition on growing axons with Nogo receptors needs to be analyzed. However, even though Rtn4b is expressed in fish oligodendrocytes (ER), the present staining with Rtn4b AB in MBP-rich regions of the normal and regenerating optic nerve is relatively weak, particularly when compared to Rtn4a AB. Rtn4a consists mainly of the reticulon homology domain (RHD) and a short N-terminal sequence but the long N-terminal region of Rtn4b with homology to the mammalian Nogo-A-specific region is absent from Rtn4a [15]. Unlike mammalian Nogo-66, zebrafish Nogo-66 within the RHD of Rtn4a is not inhibitory to axon growth but rather seems to promote growth [18].

Rtn4b is significantly upregulated in axotomized RGCs and regenerating RGC axons. Strikingly, the regenerating Neurolin-positive axons were amidst optic nerve myelin [33] consistent with the notion that fish CNS myelin is not or by far less inhibitory than mammalian CNS myelin [5,6].

## Conclusions

Notwithstanding the inhibitory activity of surface-exposed mammalian Nogo-A, our study has demonstrated that neuron-intrinsic Rtn4b/zebrafish Nogo is upregulated after optic nerve lesion in RGCs and contributes to axon regeneration as an important ER-associated factor. Robust upregulation of RTN4-A/Nogo-A and growth of the ER in mammalian RGCs concomitant with a decrease of RTN-4/Nogo-A in optic nerve myelin might increase the RGC’s competence for regeneration.

## Methods

### Animals, ONS, and morpholino application

Zebrafish were maintained at 28°C in the animal facility (TFA) of the University of Konstanz. For *in vivo* knockdown of the Rtn4b protein, the optic nerves of zebrafish, male and female, aged 4 to 8 months, were severed under 3-aminobenzoic ethylester anesthesia (MS222, 250 mg/l; Sigma-Aldrich, St. Louis, MO, USA) in compliance with animal welfare legislation. Procedures were approved by the ethical approval committee of the Regierungspräsidium Freiburg, Germany: AKZ: 35–9185.81/G-13/103. For the *in vivo* regeneration assay (see below), the ventral-most fascicles of the optic nerve which carry axons from the young peripheral RGCs were spared. A piece of gelfoam soaked in 2.5 μl of a MO solution of a concentration of either 70 or 140 μM in Ringer’s solution was applied to the proximal stump directly after ONS [17]. Two different MOs against Rtn4b [16] were used: 5′-ccactgcgggagaactcagaacagc-3′ (position on mRNA −81/−57, rtn4b MO1) and 5′ -gctcgttctgtgtcctccatcggga-3′ (position on mRNA −5/20, rtn4b MO2). The control MO sequence was 5′-aacgaacgaacgaacgaacgaacgc-3′ (absent from the zebrafish genome). All MOs (Gene Tools, Philomath, OR, USA) were labeled with lissamine and visualized in RGCs after retrograde transport. There is no noticeable RGC death following ONS in goldfish and zebrafish [34,35].

### Quantitative outgrowth assay

Five days after ONS and MO application, retinae were freed from blood vessel layer and pigment epithelium and chopped into approximately 300 × 300 μm squares. These were cultured in a poly-L-lysine (pLys)-coated 48-well plate (Greiner, Monroe, NC, USA) in L15 medium, 25 mM Hepes, 2 mM l-glutamine, penicillin (10 U/ml), streptomycin (10 μg/ml), 1% FCS, and 1 μg/l bFGF at 28.5°C. After 24 h of incubation, the number of axons per mini-explant was counted under phase contrast optics (Axiovert 35 microscope; Carl Zeiss Inc., Jena, Germany). For each MO and concentration, the experiment was performed three times with *n*, 80 explants per experimental group.

### Quantitative *in vivo* regeneration assay

To assess the regeneration capacities of RGCs under rtn4b knockdown *in vivo*, the optic nerve of MO-treated zebrafish was re-sectioned 7 days later, 2 to 3 mm distal to the first lesion, and Alexa488-dextran (Invitrogen, Carlsbad, CA, USA) was applied on the second lesion in order to retrogradely label RGCs that had regenerated their axons. At this time, the previously spared fascicles were also severed and served as control for the successful retrograde transport of the dye. After 48 h, the number of dextran-labeled RGCs was counted in retina whole mounts of control and Rtn4b knockdown retinae [17]. The experiment was performed four times with each Rtn4b and CoMO. Per experiment, *n* = 10 images were evaluated for each group. Results were statistically evaluated using Student’s *T*-test.

### Immunostaining of retina whole mounts and cryosections

For retina whole mount stainings, retinae were prepared as described above, fixed in 4% paraformaldehyde (PFA) in phosphate-buffered saline (PBS) at RT for 30 min, permeabilized by incubation in 1% Triton in PBS for 1 min at RT and exposed to immunoaffinity purified polyclonal K1121 against Rtn4b [16] 1:500 and anti-MBP 1:100 (kindly provided by William S. Talbot, Stanford University, USA), diluted in 1% BSA/PBS, overnight at 4°C. Nuclei were stained with 4′,6-diamidino-2-phenylindole (DAPI) (100 ng/ml) and cells with Alexa488-coupled Phalloidin (Invitrogen, Carlsbad, CA, USA), applied together with the secondary AB Cy3-coupled donkey anti-rabbit or Alexa488-coupled goat anti-rabbit (Jackson ImmunoResearch, West Groove, PA, USA) for 2 h at RT. For cryosections, the eye and optic nerve were isolated, transferred directly into TissueTek (Sakura, Alphen aan den Rijn, The Netherlands ) at −20°C and cut on a cryostat. The 10-μm-thick sections were transferred to pLys-coated slides and allowed to dry and either stored at −20°C or subjected directly to immunostainings with Rtn4b AB, anti MBP, IK964 against Rtn4a, or anti-neurolin (N518) against growing axons [19]. After washes in PBS, sections were coverslipped with Mowiol (Calbiochem, San Diego, CA, USA). Images were acquired at a confocal laser-scanning microscope (LSM700 META; Carl Zeiss Inc., Oberkochen, Germany) with an Apochromat 63×/1.4 oil immersion lens. For quantitative analysis of RGC size, Rtn4b-labeled cells were encircled in retinae at 5 days after ONS and normal control retinae at the LSM, evaluated by ImageJ. Fluorescence mean intensities of Rtn4b staining in control and ONS retinae were scored in three separate experiments for 12 images per group using ImageJ. Zebrafish oligodendrocytes were obtained (as described [5]) from the regenerating optic nerve/tract by explanting pieces of tissue between two coverslips in the same medium as retina explants. Cells emigrate from the nerve/tract explants, and some divide over 10 to 14 days *in vitro*. Oligodendrocytes and RGC axons were immunolabeled with Rtn4a AB, Rtn4b AB, or CLIMP63 (kindly provided by Hesso Farhan, University of Konstanz, Germany) after PFA fixation or exposed to Rtn4b AB live, then fixed and exposed to secondary ABs.

### Western blots

For Western blot analysis, isolated retinae were lysed in Ripa buffer. Blots were exposed to Rtn4b AB (diluted 1:1,000) and anti-α-Tubulin AB. The intensity of protein bands was determined by ImageJ, the Rtn4b band normalized to the loading control and statistically evaluated using the Student’s *T* test. Blots for evaluation of upregulation of Rtn4b after nerve transection were repeated four times for 5 and 10 days ONS; experiments for assessing downregulation by MO were performed five times with two retinae used per lysate.

## Abbreviations

AAV: adeno-associated virus
AB: antibody
CNS: central nervous system
Co: Control
DAPI: 4′6-diamidino-2-phenylindole
ER: endoplasmic reticulum
MO: morpholino
MS222: 3-aminobenzoic ethylester
ONS: optic nerve section
PBS: phosphate-buffered saline
PDI: protein disulfide isomerase
PFA: paraformaldehyde
RGCs: retinal ganglion cells
RHD: reticulon homology domain
RTN: reticulon
